# Exploring the Insecticidal Potential of Boldo (*Peumus boldus*) Essential Oil: Toxicity to Pests and Vectors and Non-target Impact on the Microcrustacean *Daphnia magna*

**DOI:** 10.3390/molecules24050879

**Published:** 2019-03-01

**Authors:** Roman Pavela, Giovanni Benelli, Riccardo Petrelli, Loredana Cappellacci, Giulio Lupidi, Stefania Sut, Stefano Dall’Acqua, Filippo Maggi

**Affiliations:** Crop Research Institute, Drnovska 507, 161 06 Prague, Czech Republic; pavela@vurv.cz; Department of Plant Protection, Czech University of Life Sciences Prague, Kamycka 129, 165 00 Praha 6-Suchdol, Czech Republic; Department of Agriculture, Food and Environment, University of Pisa, via del Borghetto 80, 56124 Pisa, Italy; giovanni.benelli@unipi.it; School of Pharmacy, University of Camerino, Via S. Agostino 1, 62032 Camerino Italy; riccardo.petrelli@unicam.it (R.P.); loredana.cappellacci@unicam.it (L.C.); filippo.maggi@unicam.it (F.M.); Department of Agronomy, Food, Natural Resources, Animals and Environment (DAFNAE), Agripolis Campus, University of Padova, 35020 Legnaro, Italy; Stefania_sut@hotmail.it; Department of Pharmaceutical and Pharmacological Sciences, University of Padova, Via Marzolo, 35121 Padova, Italy

**Keywords:** aquatic ecotoxicology, *Culex quinquefasciatus*, ascaridole, insecticide, *Musca domestica*

## Abstract

Every year Chile exports about 2000 tons of boldo folium (*Peumus boldus*), which is used around the world as a traditional herbal medicinal product (THMP), mostly to relieve gastrointestinal disorders. This biomass may be a resource for the agrochemical industry to manufacture botanical insecticides. In this regard, the insecticidal potential of boldo has been poorly investigated. In the present work, hydrodistillation of a commercial boldo folium gave 1.5% (*w*/*w*) of a yellowish essential oil (boldo essential oil, BEO) containing 1,8-cineole (20.7%), *p*-cymene (18.5%), limonene (9.1%), ascaridole (9.1%) and β-phellandrene (6.4%) as the main constituents, as determined by gas chromatography-mass spectrometry (GC-MS). NMR analysis allowed us to determine that ascaridole was mainly represented by the *cis*-isomer. BEO was toxic to larvae of the filariasis vector *Culex quinquefasciatus* and adults of the housefly *Musca domestica*, showing LC_50_/LD_50_ values of 67.9 mg*·*L^−1^ and 98.5 µg*·*adult^−1^, respectively. On the other hand, lower insecticidal activity was observed against larvae of the moth pest *Spodoptera littoralis* (LD_50_ of 268.9 µg*·*larva^−1^). It is worth noting that, when tested at LC_90_ concentration, BEO was significantly less toxic to aquatic microcrustacean *Daphnia magna* than the conventional insecticide α-cypermethrin. Finally, in the attempt to explore the BEO mode of action, we tested it for acetylcholinesterase (AChE) inhibitory properties using the Ellman method, obtaining negligible effects (IC_50_ = 0.45 mg·mL^−1^). Taken together, these results gave new insights into the potential of BEO as a future ingredient of botanical insecticides.

## 1. Introduction

Boldo (*Peumus boldus* Molina) is an evergreen tree belonging to the Monimiaceae family and native to temperate regions of Chile. It is a dioecious, small tree, up to 6 m tall, having an olive-like habitus, occurring as a single plant or in forests between the flat areas and the coastal cordilleras [1]. The part of this species that is used is the leaf (boldo folium), which is simple, ovate, greyish-green on the upper side and whitish on the lower, shortly petioled, hard and with a pleasant smell. The hallmark of boldo leaf is the presence of numerous bumps, corresponding to star-arranged, protective hairs, and the revolute margin [2]. The spongy mesophyll is full of oil glands producing a brownish-yellow oil (yield of 1–2%) of camphoraceous odor, aromatic taste, containing 1,8-cineole, *p*-cymene and ascaridole as the major components [3]. Notably, the latter is matter of concern due to its toxicity so that ascaridole-containing boldo preparations should be managed carefully for human use [4].

Chile exports about 2000 tons per year of *P. boldus* leaves, which are employed for the treatment of digestive and hepatic disorders, as stomachic, choleretic, cholagogue and laxative [1,5]. Notably, boldo leaf is considered a traditional herbal medicinal product (THMP) approved by European Medicines Agency (EMA), European Scientific Cooperative on Phytotherapy (ESCOP) and World Health Organization (WHO) and indicated for the relief of dyspepsia and spasmodic disorders of the gastrointestinal tract [6].

Regarding boldo essential oil (BEO), it has recently been applied, after microencapsulation, for the preservation of various commodities (e.g., peanuts) from fungal spoilage [7,8,9]. Also, important uses as a herbicide are recorded [10,11]. However, it is important to note that BEO with low levels of ascaridole is preferred from a safety perspective. Recently, there has been increasing interest in a possible usage of BEO as an active ingredient in botanical insecticides [12,13,14].

Developing novel and effective pesticides is a major challenge today, to allow the effective and eco-friendly management of arthropod pests [15,16]. In the present work, we evaluated the toxicity of BEO on larvae of the filariasis, *Culex quinquefasciatus* Say, adults of the housefly, *Musca domestica* L. and larvae of the noctuid moth *Spodoptera littoralis* (Boisduval). In addition, its impact on non-target organisms, such as the aquatic microcrustacean *Daphnia magna* Straus, was assessed. The chemical composition of BEO was obtained by gas chromatography-mass spectrometry (GC-MS) and the isomer of ascaridole was ascertained by ^1^H- and ^13^C-NMR spectroscopy.

## 2. Results

### 2.1. Composition of BEO

**Figure 1:**
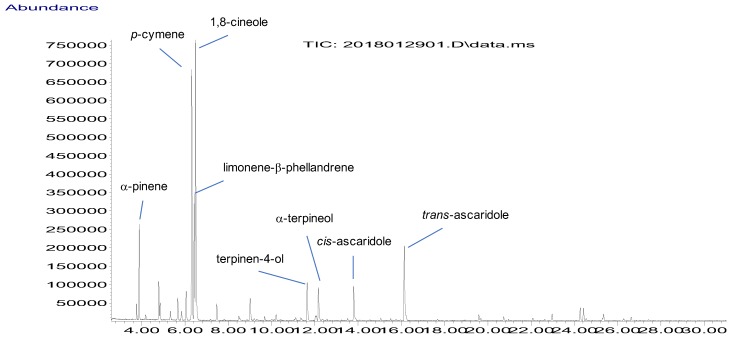
Gas chromatography-mass spectrometry (GC-MS) chromatogram of *Peumus boldus* leaf essential oil.

Figure 1 reports the chemical profile of BEO, where a total of 67 components, accounting for 98.9% of the whole composition, were identified by GC-MS, using two columns of different polarity for separation (i.e. HP-5MS and DB-WAX). The oil was dominated by monoterpenoids (50 compounds identified, 93.8%), with hydrocarbons (51.4%) prevailing on oxygen-containing compounds (42.4%) (Table 1). Within these groups, the major components were 1,8-cineole (20.7%), *p*-cymene (18.5%), limonene (9.1%), ascaridole (as sum of the two isomers *cis*- and *trans*-, 9.1%) and β-phellandrene (6.4%). Other monoterpenoids occurring in appreciable amounts (≥2%) were α-pinene (4.9%), terpinen-4-ol (3.1%), α-terpineol (2.9%), sabinene (2.4%) and α-terpinene (2.0%). The sesquiterpene fraction was rather poor (16 compounds identified, 4.6%), with any compound exceeding 1%.

### 2.2. Insecticidal Efficacy of BEO and Impact on Non-target Organisms

The present study was focused on the evaluation of the insecticidal efficacy of BEO against three target insects, namely *Cx*. *quinquefasciatus*, *M*. *domestica* and *S*. *littoralis*. Results are reported in Table 2, where noteworthy values of toxicity were obtained mostly on third instar larvae of *Cx*. *quinquefasciatus* (LC_50_ and LC_90_ values of 67.9 and 97.2 mg·L^−1^, respectively) and adults of *M*. *domestica* (LD_50_ and LD_90_ values of 98.5 and 173.9 mg·adult^−1^, respectively). Significantly lower values were obtained with α-cypermethrin used as positive control (LC_50(90)_ of 0.008(0.025) mg·L^−1^ on *Cx*. *quinquefasciatus*; LD_50(90)_ of 0.16(0.85) mg·adult^−1^ on *M*. *domestica*). Besides, the toxicity of BEO against larvae of the agricultural pest *S*. *littoralis* was moderate, showing LD_50(90)_ of 268.9(556.9) μg·larva^−1^ (Table 2).

In the evaluation of the activity of BEO, its potential effects on non-target aquatic species were also considered. *Daphnia magna* is an aquatic microcrustacean rather sensitive to conventional insecticides and is frequently used to evaluate the ecotoxicological effects of new products [17]. As a matter of fact, the positive control used in our experiments, that is, α-cypermethrin, caused a 100% mortality at both 24 and 48 h when tested at its LC_90_ estimated on *Cx*. *quinquefasciatus* larvae (0.025 mg·L^−1^) (Table 3). In this respect, BEO, tested at 96.2 mg·L^−1^ produced a significantly lower toxicity on the microcrustacean (46.2 and 66.2% after 24 and 48 h from treatments, respectively) (Table 3).

### 2.3. Inhibitory Properties of BEO on Acetylcholinesterase (AChE)

To shed light on the possible mode of action of BEO, we assayed its inhibitory properties against AChE, which is an important target for testing the insecticidal potential of synthetic and natural products. In our assay, the BEO half-inhibited AChE at a concentration of 0.45 mg·mL^−1^ (Table 4). This IC_50_ value was about 56-times higher than that of galanthamine used as positive control and corresponded to 17.97 mgGEIC·gr^−1^.

## 3. Discussion

The hallmark of the BEO analyzed in this study was a lower amount of ascaridole (9.1%) compared to those reported in earlier studies by Petigny et al. [18], Herrera-Rodriguez et al. [19], Blázquez and Carbó [11], de Castro et al. [12], namely 46.9, 24.4, 38.9 and 31.4%, respectively. On the other hand, in the research by Passone and Etcheverry [9] ascaridole was not detected at all (Table 5). Besides, in the above-mentioned studies there was consistency for the other major components of BEO, namely 1,8-cineole and *p*-cymene if compared to earlier researches summarized in Table 5.

**Figure 2:**
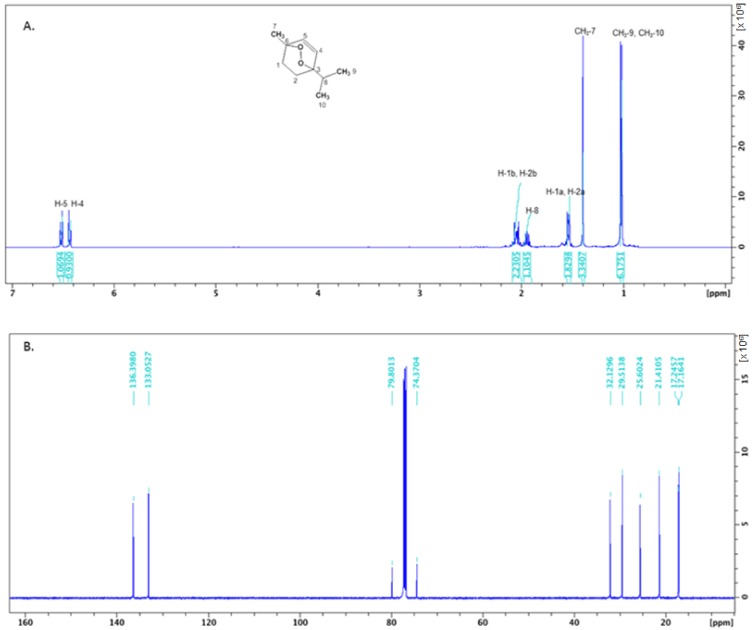
^1^H- (**A**) and ^13^C-NMR (**B**) spectrum (500 MHz) of *cis*-ascaridole from *Peumus boldus* leaf essential oil.

Ascaridole is a bicyclic monoterpene endowed with a 1,4-endoperoxide ring formed from α-terpinene [20]. The most abundant form occurring in nature is the *cis*-isomer, which, under thermal rearrangement, is converted into the *trans*-isomer [21]. The identification of the *cis* form in our study was carried out by NMR analysis after purification by column chromatography (Figure 2). The study of NMR spectra showing only the *cis* form suggests that the *trans* form is an artifact produced during the GC run. This conversion can occur during vaporization in the GC instrument injection liner, or during the thermal ramp in the chromatographic separation. The biological activity of ascaridole is linked to the breakdown of the peroxide group leading to the formation of radicals which are toxic to several parasites (e.g., *Plasmodium falciparum*) and insect pests (e.g., stored product beetles) [22,23].

In our experiments, the insecticidal activity exhibited by BEO may be linked to the bioactivity of its major components, with special reference to 1,8-cineole, *p*-cymene, limonene and ascaridole. Ascaridole is also a chemical marker of the essential oils from *Dysphanya ambrosioides* (L.) Mosyakin & Clemants and *Ledum palustre* L. [24,25], which displayed notable toxicity on the same insect species. Notably, the oil from *D. ambrosioides*, containing higher levels of ascaridole (61.4%), showed LC_50_ of 62.1 ppm on *Cx. quinquefasciatus,* which was similar to that of BEO (67.9 mg·L^−1^). On the other hand, it was more toxic to adults of *M. domestica* (LD_50_ of 51.7 μg·adult^−1^) when compared to BEO (98.5 μg·adult^−1^). We assume that the higher concentration of ascaridole, along with the lower amount of the insecticidal *p*-cymene [26] and the presence of other minor components, might explain the bioactivity differences detected between these two oils. Besides, the ascaridole-poor BEO tested here, can induce higher toxicity on the third instar larvae of *Cx. quinquefasciatus* when compared with that analyzed by de Castro et al. [12] (containing 31.4% ascaridole), who obtained an LC_50_ value of 82 ppm on the above-mentioned mosquito species. The same authors found an increase in toxicity in the ascaridole-enriched fraction (LC_50_ of 41.9 ppm). They correlated the toxicity of this ascaridole-rich essential oil to its capacity to induce midgut damages in the larvae [12].

It is worth noting that in mammals ascaridole is rather toxic, as its LD_50_, determined in rats after oral administration is 130 mg·kg^−1^ [27]. Thus, the usage of this compound and ascaridole-rich essential oils should be treated carefully.

1,8-Cineole is a monoterpene ether which is typical of many aromatic plants, mostly *Eucalyptus* spp. and Mediterranean bay (*Laurus nobilis* L.), where it is responsible for the antiseptic, expectorant and mucolytic properties and thus used for the treatment of respiratory diseases [28,29]. 1,8-Cineole is considered one of the most important mosquito repellents produced by aromatic plants [30]. It also occurs in several essential oils endowed with noteworthy larvicidal effects (LC_50_ values below 50 ppm) [31]. Notably, 1,8-cineole is an ingredient of Eco-oil^®^, a natural product used in Australia against insects and mites [32]. From a toxicological perspective 1,8-cineole is relatively safe, having an LD_50_ value of 2480 mg·kg^−1^ in rats [33,34]. It is also recognized as a generally recognized as safe (GRAS) substance [35].

*p*-Cymene is an aromatic monoterpene occurring in high levels in the essential oil from *Schizogyne sericea* (L.f.) DC [36] and functions as precursors of phenolics as thymol and carvacrol [37,38,39,40]. It is a strong perturbator of cell membrane and can synergize the effects of other active compounds, such as ascaridole [26,38,39,40].

Limonene is a ubiquitous monocyclic monoterpene having LC_50_ values below 20 ppm on various mosquitoes, including *Anopheles stephensi* Liston, *Aedes aegypti* L., *Ae. albopictus* (Skuse) and *Cx. quinquefasciatus* [31]. This compound can interact with octopamine receptors and alters the nerve transmission and coordination in various insects [31]. Limonene is a GRAS substance and with low toxicity to mammals (LD_50_ of 4600 mg·kg^−1^ in rats) [33,34,41]. Since 2014, limonene has been used to prepare botanical insecticides like Prev-Am^®^ [41,42,43].

Thus, the insecticidal effects exhibited by BEO in this study could be the result of the complex interactions between its major components. Interestingly, the toxicity of BEO on housefly adults observed by us supported earlier findings by Urzua et al. [44] although the experimental procedure followed by these authors was rather different. Furthermore, the BEO analyzed by these authors presented similar composition compared with our data, showing low levels of ascaridole (6.3%) and high percentages of 1,8-cineole (36.6%) and *p*-cymene (29.8%) [44].

Regarding the possible mode of action, our experiments conducted on the target AChE testing BEO indicated only a negligible activity (IC_50_ = 450 μg·mL^−1^). This was very low when compared with that exhibited by *D. ambrosioides* (IC_50_ = 77 μg·mL^−1^) [24] and *Origanum compactum* Benth. (IC_50_ = 124 μg·mL^−1^) [45] essential oils, but higher than that of *Crithmum maritimum* L. [46] (IC_50_ > 3500 μg·mL^−1^) and *Cannabis sativa* L. (IC_50_ = 4000 μg·mL^−1^) [47] essential oils. These findings suggest alternative ways through which BEO exert toxicity in the targeted insects. As an example, monoterpenoids occurring in BEO (e.g., *p*-cymene, limonene, phellandrene, 1,8-cineole) may be inhibitors of GABA-gated chloride channels in insects [48] and/or bind to octopamine receptors that are involved in important physiological functions of insects [49]. Besides, as reported above, ascaridole may induce oxidative stress in insects through the production of toxic radicals [22,23]. Thus, this interesting line of research is expected to be pursued in the years to come in order to better understand the potentiality of plant-borne essential oils.

## 4. Materials and Methods

### 4.1. Plant Material

Dry leaves of *P. boldus* (1 kg) were kindly provided from A. Minardi & Figli S.r.l. (batch n. MP230617231117, www.minardierbe.it) in March 2018 and came from spontaneous trees of Chile (collection 2017).

### 4.2. Hydrodistillation of Essential Oil

Boldo leaves (1 kg) were coarsely crushed, then inserted into a 10 L flask and 6 L of deionized water was added. Afterwards, the Clevenger-type apparatus was inserted at flask’s neck and hydrodistillation has begun. After 3 h a yellowish oil was collected and stored in dark vials sealed with PTFE-silicon caps (Sigma-Aldrich, Milan, Italy). The oil yield was determined on a dry weight basis; it was 1.8%.

### 4.3. GC-MS Analysis

An Agilent 6890N gas chromatograph equipped with a 5973N mass spectrometer was used for the determination of BEO chemical composition. A 5% phenylmethylpolysiloxane HP-5MS (30 m × 0.25 mm i.d. × 0.1 μm film thickness, Agilent, Folsom, CA, USA) and a polyethylene glycol DB-WAX (30 m × 0.25 mm i.d. × 0.25 μm film thickness, Agilent) were used as stationary phases. Helium (99.99%) was the mobile phase with a flow rate of 1.4 mL·min^−1^. The operative conditions (e.g., oven temperature, split ratio, injector and detector temperatures, software for data processing etc.) adopted were the same of those reported by Benelli et al. [50,51] and Quassinti et al. [52]. Two μL of the hexanic solution of essential oil (6 μL of essential oil in 594 μL of solvent) were injected three times and analyzed in full scan (29–400 *m*/*z*) using the electron impact (EI) mode. The peak assignment was made by using three different approaches: (i) correspondence of the RI, calculated using a mix of *n*-alkanes (C8–C20 purchased from Supelco, Bellefonte, CA, USA), with respect to those reported in ADAMS [53], NIST 17 [54] and FFNSC2 [55] libraries for apolar and polar columns; (ii) MS matching with the WILEY275, ADAMS, NIST 17 and FFNSC2 libraries; (iii) co-injection with available analytical standard (see Table 1). Relative abundances of peaks were obtained by peak area normalization without using response factors.

### 4.4. Isolation and Identification of cis-Ascaridole

0.5 mL of *P. boldus* essential oil (approximatively 400 mg) were chromatographed on a silica gel (20 g) column (70−230 mesh, 60 Å, Merck, Kenilworth, NJ, USA) using cyclohexane, followed by a stepwise gradient solvent system consisting of cyclohexane/ethyl acetate 99:1 to 97:3. A total of 85 fractions (3 mL) were collected and monitored by thin-layer chromatography (TLC). Fractions 65-74 (AS65-74) yielded 120 mg of a pure compound, and the structure was identified as *cis*-ascaridole. Both samples, that is, essential oil and AS65-74, were found to contain a certain amount of *trans*-ascaridole when analyzed by GC/MS analysis, but ^1^H- and ^13^C-NMR spectral data of AS65-74 showed only signals assigned to *cis*-ascaridole. The *trans*-ascaridole contents in the essential oil sample and AS65-74 were considered to be artifacts produced during the GC-MS analysis [21]. ^1^H- NMR and ^13^C-NMR spectra were recorded on 500 MHz NMR spectrometer (Bruker Avance III 500 MHz, Bruker, Billerica, MA, USA). The chemical shift values are expressed in δ values (ppm), and coupling constants (*J*) are in hertz; tetramethylsilane (TMS) was used as an internal standard. Proton chemical data are reported as follows: chemical shift, multiplicity (s = singlet, d = doublet, dd = doublet of doublets, t = triplet, dt = doublet of triplets, q = quartet, sept = septet, m = multiplet, brs = broad singlet) coupling constant (s), integration. The NMR data were compared with those reported in the literature, allowing it to be identified as *cis*-ascaridole [21].

^1^H-NMR (CDCl_3_-*d*_6_): δ 1.03 (d, *J* = 6.9, 6H, H9, H10), 1.39 (brs, 3H, H7), 1.54 (d, *J* = 10.6, 2H, H1b, H2b),1.94 (sept, *J* = 6.9, 1H, H8), 2.02–2.07 (m, 2H, H1a, H2a), 6.43 (d, *J* = 8.7, 1H, H5), 6.52 (d, *J* = 8.7, 1H, H4). ^13^C-NMR (DMSO-*d*_6_): 17.16 (isopropyl CH_3_), 17.25 (isopropyl CH_3_), 21.41 (CH_3_-7), 25.61 (C-1), 29.51 (C-2), 32.13 (isopropyl C), 74.37 (C-6), 79.81 (C-3), 133.05 (C-5), 136.39 (C-4). MS (API-ESI): *m*/*z* 169.12 [M + H]^+^. Anal. calcd. for (C_10_H_16_O_2_) C, 71.39; H, 9.59; Found: C, 71.38; H, 9.61.

### 4.5. Toxicity against Culex quinquefasciatus Larvae

The acute toxicity, measured as mortality after 24 h of exposure, was determined on 3rd instar larvae of *Cx. quinquefasciatus*. Mosquito larvicidal assays were carried out according to WHO Standard Procedures, with slight modifications [26]. For experimental treatment, 1 mL DMSO (Sigma-Aldrich, Darmstadt, Germany) of serial dilutions of BEO (10, 25, 50, 70, 80, 90 and 100 mg·L^−1^) was added to 224 mL of distilled water in a 500-mL glass bowl and shaken lightly to ensure a homogenous test solution. The selected larvae (25 larvae per concentration, 4 replicates) were transferred in water into a bowl of prepared test solution with a final surface area of 125 cm^−2^. Distilled water containing the same amount of DMSO used to test EO was used as negative control and α-cypermethrin (Vaztak®) was used as the positive control (concentrations: 0.001, 0.003, 0.005, 0.008, 0.01, 0.02 and 0.03 mg·L^−1^). After 24 h treatment mortality was determined. Laboratory conditions were 25 ± 1 °C, 70 ± 3% R.H. and 16:8 h (L:D) during both rearing and toxicity tests.

### 4.6. Toxicity against Musca domestica Adults

The acute toxicity, measured as mortality after 24 h of exposure, was determined by topical application on *M. domestica* adult females (2–5 days old) [56]. Houseflies, anesthetized with CO_2_, were treated with 1 μL of acetone (Sigma-Aldrich, Prague, Czech Republic) containing BEO at doses of 30, 50, 100, 130, 160, 180 and 200 μg·adult^−1^ (20 adults per concentration, 4 replicates) using a microelectric applicator. Acetone was the negative control and α-cypermethrin (Vaztak®) at 0.05, 0.1, 0.2, 0.3, 0.5, 0.7, 0.9 and 1.2 μg·adult^−1^ was used as the positive control. Post-treatment, females were transferred to a recovery box (10 × 10 × 12 cm) for 24 h and mortality was determined. Laboratory conditions were 25 ± 1 °C, 70 ± 3% R.H. and 16:8 h (L:D) during both rearing and toxicity tests.

### 4.7. Larval Toxicity on Spodoptera littoralis

The acute toxicity of BEO and α-cypermethrin, measured as mortality after 24 h of exposure, was determined by topical application on larvae (weight ranging from 20 to 25 mg) of *S. littoralis* (3rd instar) following our earlier method [57]. Boldo EO was diluted in 1 μL of acetone at doses of 100, 150, 200, 250, 300, 350, 400, 450 and 500 μg·larva^−1^. Four groups, each composed of 20 larvae, were tested for each dose. Acetone was the negative control and α-cypermethrin (Vaztak®) at 0.005, 0.008, 0.01, 0.015 and 0.02 μg·larva^−1^ was used as the positive control. After treatment, larvae were transferred to a recovery box (10 × 10 × 7 cm) for 24 h for mortality determination. Laboratory conditions were 25 ± 1 °C, 70 ± 3% R.H. and 16:8 h (L:D) during both rearing and toxicity tests. Death was recorded when the larvae did not respond to prodding with forceps.

### 4.8. Impact on Non-target Microcrustaceans

The acute toxicity tests were carried out in accordance with the guidelines of the OECD with slight modifications [58,59]. Laboratory-reared *D. magna* adults (2–5 days old) were used as a non-target organisms to assess the impact of BEO on aquatic invertebrates, also testing α-cypermethrin as positive control. Twenty *D. magna* were exposed in each test vessel and four replicates were carried out, for a total of 80 *D. magna* per treatment. *D. magna* individuals were placed in containers with 250 mL of pure water, and BEO or α-cypermethrin was then mixed into the water at concentrations corresponding to the LC_90_ estimated on *Cx. quinquefasciatus* larvae (for EO = 96 mg·L^−1^ and for α-cypermethrin = 0.025 mg·L^−1^). The chemicals were emulsified using DMSO (in the ratio 1:1). Mortality was calculated after 24 and 48 h. Laboratory conditions were 25 ± 1 °C, 70 ± 3% R.H. and 16:8 h (L:D) during both rearing and toxicity tests.

### 4.9. Acetylcholinesterase (AChE) Inhibitory Activity

Inhibition of AChE activity of BEO was measured following the method of Ellmann with slight modifications [46]. Briefly, aliquots of BEO diluted in methanol were mixed with 50 μL of 50 mM phosphate buffer pH 8, 125 μL of dithionitrobenzoic acid (DTNB, 3 mM in 50 mM phosphate buffer pH 8) and 25 μL of AChE from electric eel, 3 U/mL in 50 mM phosphate buffer pH 8) and incubated for 15 min at 25 °C. Then, 25 μL of the substrate acetylthiocholine iodide (ACTI, 15 mM in 50 mM phosphate buffer pH 7.0) were added and AChE activity was calculated by measuring the absorbance at 412 nm (at 25 °C for 3.0 min) with a Fluostar Omega (BMG-Labtech, Ortenberg, Germany) plate reader. Galantamine (Sigma, Milan, Italy) was used as a reference substance. Results were expressed as galantamine-equivalent inhibition capacity (GEIC), indicating the mg of galantamine equivalents (GE) per g of BEO. Each experiment was replicated three times.

### 4.10. Statistical Analysis

When control mortality did not exceed 20%, the Abbott’s formula [60] was applied to correct experimental mortality rates. If control mortality was >20%, experiments were not considered and repeated. LD_50(90)_ or LC_50(90)_ for the targeted organisms, with associated 95% CL and chi-square values, were calculated using probit analysis [61]. The concentration of BEO causing 50% AChE inhibition (IC_50_) was calculated via nonlinear regression analysis.

## 5. Conclusions

Taken together, our findings indicate that the analyzed BEO can be considered a relatively safe pest and vector control product, due to the low content of ascaridole (toxic to vertebrates), thus presenting a potential use in the area of botanical insecticide development. Starting from the present data showing low inhibition of AChE triggered by BEO, further studies are necessary to shed light on its modes of action, with special reference to the potential inhibition of GABA-gated chloride channels and/or binding to octopamine receptors, as well as to assess its efficacy on other target insects of agricultural and public health importance. Besides, its safety towards other beneficial organisms still needs to be elucidated in real-world conditions, to ascertain the eco-friendliness of this natural product and its potential as ingredient in botanical insecticides. Its scalability on an industrial level may be assured by the huge biomass of boldo folium, which is exported all around the world and used to make pharmaceutics and herbal remedies.

## Figures and Tables

**Table 1:** Chemical composition of the essential oil obtained from the leaves of *Peumus boldus*.

No	Component ^a^	RI Apolar Column		RI Polar Column		% ^f^	ID ^g^
		Exp. ^b^	Lit. ^c^	Exp. ^d^	Lit. ^e^		
1	α-thujene	921	924			0.8 ± 0.1	1,2
2	α-pinene	927	932	1020	1022	4.9 ± 0.9	1,2,3
3	Camphene	940	946	1063	1066	0.2 ± 0.0	1,2,3
4	Sabinene	966	969	1118	1120	2.4 ± 0.5	1,2,3
5	β-pinene	969	974	1107	1110	1.1 ± 0.2	1,2,3
6	Myrcene	990	988	1156	1156	0.6 ± 0.2	1,2,3
7	dehydro-1,8-cineole	990	988	1192	1195	0.6 ± 0.2	1,2
8	α-phellandrene	1003	1002	1161	1161	1.6 ± 0.4	1,2,3
9	δ-3-carene	1008	1008	1145	1145	0.6 ± 0.3	1,2,3
10	α-terpinene	1015	1014	1176	1176	2.0 ± 0.4	1,2,3
11	*p*-cymene	1022	1020	1266	1267	18.5 ± 2.1	1,2,3
12	Limonene	1025	1024	1196	1199	9.1 ± 1.6	1,2,3
13	β-phellandrene	1025	1025	1206	1206	6.4 ± 1.2	1,2,3
14	1,8-cineole	1027	1026	1213	1212	20.7 ± 3.1	1,2,3
15	(*E*)-β-ocimene	1047	1044	1246	1246	0.1 ± 0.0	1,2,3
16	γ-terpinene	1056	1054	1242	1244	1.2 ± 0.3	1,2,3
17	*cis*-sabinene hydrate	1065	1065	1469	1469	0.1 ± 0.0	1,2
18	Terpinolene	1085	1086	1279	1278	0.3 ± 0.1	1,2,3
19	Fenchone	1085	1083	1400		0.3 ± 0.1	1,2
20	*p*-cymenene	1087	1089	1432	1432	0.1 ± 0.0	1,2
21	*trans*-sabinene hydrate	1097	1098	1555		0.1 ± 0.0	1,2
22	Linalool	1101	1095	1545	1545	1.9 ± 0.4	1,2,3
23	1,3,8-*p*-menthatriene	1109	1108	1390		0.1 ± 0.0	1,2
24	*trans*-*p*-mentha-2,8-dien-1-ol	1119	1119	1633	1637	0.3 ± 0.1	1,2
25	*trans*-pinocarveol	1134	1135	1664	1664	0.5 ± 0.1	1,2
26	*trans*-*p*-menth-2-en-1-ol	1138	1136			0.1 ± 0.0	1,2
27	Camphor	1140	1141	1522	1519	0.1 ± 0.0	1,2,3
28	sabina ketone	1156	1154	1641	1651	0.1 ± 0.0	1,2
29	Pinocarvone	1158	1160	1573		0.3 ± 0.1	1,2
30	Borneol	1161	1165			tr ^h^	1,2,3
31	δ-terpineol	1165	1162			0.4 ± 0.1	1,2
32	terpinen-4-ol	1173	1174	1606	1603	3.1 ± 0.6	1,2,3
33	Cryptone	1181	1183	1680	1679	tr	1,2
34	*trans*-*p*-mentha-1(7),8-dien-2-ol	1184	1187			0.8 ± 0.2	1,2
35	α-terpineol	1187	1186	1700	1700	2.9 ± 0.6	1,2,3
36	Myrtenal	1192	1195	1633	1634	0.3 ± 0.1	1,2,3
37	Myrtenol	1192	1194	1632		0.2 ± 0.0	1,2,3
38	*trans*-piperitol	1205	1207			tr	1,2
39	*trans*-carveol	1217	1215	1841	1840	0.1 ± 0.0	1,2
40	*cis*-*p*-mentha-1(7),8-dien-2-ol	1225	1227			0.2 ± 0.0	1,2
41	*cis*-ascaridole	1233	1234			3.0 ± 0.7	1,2,3
42	cumin aldehyde	1236	1238	1783	1781	0.2 ± 0.0	1,2
43	Carvone	1242	1239	1740	1738	0.1 ± 0.0	1,2,3
44	*trans*-piperitone epoxide	1255	1252	1734	1733	0.1 ± 0.0	1,2
45	*p*-menth-1-en-7-al	1269	1273			0.2 ± 0.0	1,2
46	bornyl acetate	1282	1287	1584	1584	0.2 ± 0.0	1,2,3
47	Thymol	1289	1289	2191	2189	0.1 ± 0.0	1,2,3
48	*trans*-ascaridole	1301	1303	1874		6.1 ± 1.1	1,2,3
49	Carvacrol	1303	1298	2203	2201	0.5 ± 0.2	1,2,3
50	α-terpinyl acetate	1347	1346	1700	1701	0.1 ± 0.0	1,2
51	β-elemene	1386	1389	1590	1591	tr	1,2,3
52	methyl eugenol	1406	1403	2007	2006	0.6 ± 0.2	1,2
53	(*E*)-caryophyllene	1409	1417	1600	1604	0.2 ± 0.0	1,2,3
54	α-humulene	1444	1452	1673	1680	0.3 ± 0.1	1,2,3
55	*allo*-aromadendrene	1451	1458	1650	1650	0.1 ± 0.0	1,2
56	bicyclogermacrene	1488	1500	1737	1735	0.2 ± 0.0	1,2
57	α-muurolene	1495	1500	1724		tr	1,2
58	γ-cadinene	1506	1513	1761	1762	0.1 ± 0.0	1,2
59	δ-cadinene	1518	1520	1757	1757	0.5 ± 0.1	1,2
60	α-calacorene	1535	1544	1917		tr	1,2
61	(*E*)-nerolidol	1563	1561	2040	2039	1.0 ± 0.2	1,2,3
62	spathulenol	1568	1577	2135	2136	1.0 ± 0.2	1,2
63	caryophyllene oxide	1572	1582	1997	1994	0.2 ± 0.0	1,2,3
64	humulene epoxide II	1598	1608	2056	2069	0.1 ± 0.0	1,2
65	β-oplopenone	1600	1607	2086	2089	0.5 ± 0.1	1,2
66	*epi*-α-muurolol	1634	1640	2191	2190	0.2 ± 0.0	1,2
67	α-cadinol	1647	1652	2242	2251	0.3 ± 0.0	1,2
	Total identified (%)					98.9	
	Monoterpene hydrocarbons					51.4	
	Oxygenated monoterpenes					42.4	
	Sesquiterpene hydrocarbons					1.4	
	Oxygenated sesquiterpenes					3.2	
	Others					0.6	

^a^ Compounds’ order is according to their elution from a HP-5MS column (5% phenylmethylpolysiloxane, 30 m × 0.25 µm i.d. × 0.1 µm f.t.). ^b^ Temperature-programmed retention index calculated on a HP-5MS column. ^c^ Retention index taken from ADAMS and NIST 17 libraries for apolar columns. ^d^ Temperature-programmed retention index calculated on a DB-WAX (polyethylene glycol, 30 m × 0.25 mm i.d. × 0.25 μm f.t.) column. ^e^ Retention index taken from NIST 17 for polar columns. ^f^ Peak area percentage (mean of three measurements) ± standard deviation. ^g^ Identification method: 1 correspondence of the RI with those reported by ADAMS, NIST 17 and FFNSC2 libraries; 2 MS matching with WILEY275, ADAMS, NIST 17 and FFNSC2 libraries; 3 correspondence of RT, RI and MS with that of analytical standard. ^h^ Percentage below 0.1%.

**Table 2:** Lethal concentrations (LC) and lethal doses (LD) of *Peumus boldus* leaf essential oil and positive control against target insects.

	Target Organisms	LC/LD_50_ ± SE	CI_95_	LC/LD_90_ ± SE	CI_95_	*χ^2^*	*d.f.*	*p-Value*
BEO	*Cx. quinquefasciatus*(larvae, 3rd instar) mg·L^−1^	67.9 ± 7.8	55.1–70.5	96.2 ± 7.9	91.5–102.3	5.253	3	0.395
	*M. domestica*(adults) µg·adult^−1^	98.5 ± 5.2	89.7–102.3	173.9 ± 10.9	165.9–198.7	1.452	3	0.985
	*S. littoralis*(larvae, 3rd instar) µg·larva^−1^	268.9 ± 12.6	252.8–295.5	556.9 ± 22.9	621.5–708.9	0.187	3	0.658
Positive controlα-cypermethrin	*Cx. quinquefasciatus* (larvae, 3rd instar) mg·L^−1^	0.008 ± 0.001	0.006–0.012	0.025 ± 0.002	0.021–0.032	5.235	3	0.296
	*M. domestica*(adults) µg·adult^−1^	0.16 ± 0.2	0.16–0.22	0.85 ± 0.1	0.82–1.12	1.525	3	0.752
	*S. littoralis*(larvae, 3rd instar) µg·larva^−1^	0.009 ± 0.003	0.007–0.012	0.021 ± 0.009	0.018–0.028	2.525	3	0.395

SE = standard error, CI_95_ = 95% confidence interval, *d.f.* = degrees of freedom.

**Table 3:** Acute toxicity of *Peumus boldus* leaf essential oil and α-cypermethrin against *Daphnia magna* adults. All compounds were tested at the LC_90_ calculated on *Culex quinquefasciatus* 3rd instar larvae.

	*Daphnia magna* Mortality (%) ^a^	
	After 24 h	After 48 h
BEO (96.2 mg·L^−1^)	46.2 ± 4.1 ^b^	66.2 ± 4.1 ^b^
α-cypermethrin (0.025 mg·L^−1^)	100.0 ± 0.0 ^c^	100.0 ± 0.0 ^d^
Negative control	0.0 ± 0.0 ^a^	0.0 ± 0.0 ^a^
ANOVA *F_2,8_; P*	1251.3; <0.0001	1338.5; <0.0001

^a^ Microcrustacean mortality was expressed as mean values (%) ± SE. Within each column, different letters indicate significant differences among values (*p* < 0.05).

**Table 4:** Acetylcholinesterase inhibitory properties of *Peumus boldus* leaf essential oil.

	IC_50_ mg·mL^−1^	mgGEIC·gr^−1 a^
BEO	0.45 ± 0.03	17.97 ± 1.0
Positive control		
Galantamine	8 ± 0.2 × 10^−3^	

^a^ GEIC = galantamine-equivalent inhibition capacity.

**Table 5:** Major components (in decrescent order) of (boldo essential oil, BEO) reported in previous studies.

Origin	Main Oil Components	Reference
Not specified	ascaridole (46.9%), limonene (18.8%), *p*-cymene (12.9%)	[16]
Chile	ascaridole (24.4%), 1,8-cineole (14.9%), (*E*)-β-ocimene (12.9%)	[17]
Commercial	ascaridole (38.9%), *p*-cymene (21.6%), 1,8-cineole (12.6%)	[11]
Commercial	ascaridole (31.4%), 1,8-cineole (25.0%), *o*-ocimene (11.7%)	[12]
Commercial	piperitone oxide (28.1%), α-terpinene (18.8%), 1,8-cineole (12.3%)	[9]
Chile	1,8-cineole (36.6%), *p*-cymene (29.8%), ascaridole (6.2%)	[44]
